# Supplementary material for: Dissipation and Distribution of Picarbutrazox Residue Following Spraying with an Unmanned Aerial Vehicle on Chinese Cabbage (Brassica campestris var. pekinensis)
Source: Molecules. 2021 Sep 18;26(18):5671. doi: 10.3390/molecules26185671 (PMC8472731; doi:10.3390/molecules26185671)
Supplement: Supplementary file 1 [file molecules-26-05671-s001.zip › molecules-1345839-SI.pdf]

Supporting Information

Dissipation and distribution of picarbutrazox residue following spraying with unmanned aerial vehicle onto Chinese cabbage (*Brassica campestris* var. *pekinensis*)

Chang Jo Kim[1,#], Won Tae Jeong[1,#], Hyun Ho Noh[1,*], Kee Sung Kyung[2], Hee-Dong Lee[1], Danbi Kim[1], Ho-Sung Song[3], and Younkoo Kang[4]

Number of pages: 9

Supplementary Methods, 5 Supplementary Tables (Tables S1–S5)

Supplementary Methods

Reagents and materials

Standards of picarbutrazox (purity > 98.8%) and its metabolite TZ-1E (purity > 99.6%) were obtained from Kyung Nong, a commercial pesticide company in Korea. Acetonitrile (high-performance liquid chromatography-grade) and formic acid (purity > 98%) were purchased from Thermo Fisher Scientific (Waltham, MA, USA). QuEChERS extraction and dispersive-solid phase extraction (d-SPE) kits were obtained from Agilent Technologies (Santa Clara, CA, USA). The shaker and centrifuge used for sample extraction were obtained from Geno/Grinder (SPEX SamplePrep, Metuchen, NJ, USA) and Combi-514R (Hanil Scientific Inc., Incheon, Korea), respectively. The syringe filter (pore size, 0.22 μm) with polytetrafluoroethylene (PTFE) was purchased from Advantec (Tokyo, Japan). Instrumental analysis with liquid chromatography-tandem mass spectrometry (LC-MS/MS) was carried out on an AB SCIEX Triple Quad™ 5500 system coupled with an Exion LC™ column (SCIEX, Redwood City, CA, USA). The C_{18} reverse-phase column (2.1 mm I.D. × 150 mm L., 2.7 μm) was obtained from Advanced Materials Technology (Wilmington, DE, USA).

Optimization of mass spectrometry

If compounds are stable at high temperatures, and contain carbonyl groups and nitrogen in their molecular structures, they can be analyzed with a gas chromatograph coupled with a nitrogen phosphorus detector or electron-capture detector[1]. Although the molecular structures of the tested pesticides contain carbonyl groups and nitrogen, they also contain amino groups that are unstable at high temperatures and start degrading at temperatures above 150 °C. Moreover, picarbutrazox and TZ-1E are volatile compounds with a vapor pressure of 1.2×10^{-7} Pa (50 °C). Accordingly, these compounds need to be analyzed using LC-MS/MS[2].

For LC, the mobile phases were 0.1% formic acid in distilled water (A) and acetonitrile (B). A Halo C_{18} reverse-phase column was used, which is suitable for separating polar and non-polar materials effectively, and the injection volume was 1 µL. The gradient elution was started at 70% mobile phase B for 2 min, which was decreased to 30% for 0.1 min, and was maintained for 5 min. For the next sample, mobile phase B was steeply increased to 70% for 0.1 min and maintained for 1.9 min to achieve equilibrium. The total run time was 6 min. The ion spray voltage was 5500 V and the source was electrospray ionization. The pressure of the nebulizer, curtain, drying, and collision gases was 50, 20, 50, and 10 psi, respectively. Multiple-reaction monitoring mode was used to improve selectivity and sensitivity. The precursor ions of picarbutrazox and TZ-1E were *m/z* 410.1, and their quantitation ion and collision energy (CE) were *m/z* 310.1 and 19 eV, respectively. The confirmation ion and CE of the analytes were *m/z* 80.1 and 71 eV, respectively.

Limit of quantitation, reproducibility, and recovery

The limit of quantitation (LOQ) was determined as the minimum concentration that provided a signal-to-noise ratio over 10 on the chromatogram. The calibration curve was obtained based on the peak area analyzed by a matrix-matched standard with LC-MS/MS, and the linearity of the calibration curve was expressed as a correlation coefficient (r^2). The recovery test of picarbutrazox and TZ-1E was performed with three replicates by analyzing three different levels: 0.005, 0.05, and 0.25 mg kg^{-1}. The recovery test result was validated according to the Food and Agriculture Organization of the United Nations (FAO)[3], in which fortification levels were verified by classification into five groups: (1) ≤0.001 mg kg^{-1}, 50%−120% recovery range, coefficient of variation (CV) ≤ 35%; (2) >0.001 and ≤0.01 mg kg^{-1}, 60%−120% recovery range, CV ≤ 30%; (3) >0.01 and ≤0.1 mg kg^{-1}, 70%−120% recovery range, CV ≤ 20%; (4) >0.1 and ≤1.0 mg kg^{-1}, 70%−110% recovery range, CV ≤

15%, and (5) >0.1 mg kg^{-1}, 70%–110% recovery range, CV ≤ 10%. Matrix-matched standards of LOQ and 10LOQ concentration were analyzed six times and the CV of peak area, height, and retention time were calculated for the reproducibility test with LC-MS/MS.

References

1. Noh, H.H.; Kim, C.H.; Kwon, H.Y.; Kim, D.B.; Moon, B.; Baek, S.; Oh, M.; Kyung, K.S. Optimized residue analysis method for broflanilide and its metabolites in agricultural produce using the QuEChERS method and LC-MS/MS. *PLOS One*. **2020**, *15* e0235526.

2. Noh, H.H.; Moon, B.C.; Kim, C.J.; Kwon, H.; Ro, J.H.; Kim, D.; Oh, M.S.; Kim, B.S.; Kim, H.T.; Kyung, K.S. Residual characteristics of picarbutrazox and its metabolite TZ-1E by aerial spraying with agricultural multi-copter in shallot. *Korean J. Pest. Sci*. **2019**, *23*(3) 162−171.

3. Food and Agriculture Organization of the United Nations (FAO). Submission and evaluation of pesticide residues data for the estimation of maximum residue levels in food and feed. Pesticide Residues, FAO, **2016**, pp. 27−32.

Table S1. Average temperature, humidity, and wind speed and direction during pesticide spraying.

Spray date	Plot	Temperature (℃)	Humidity (%)	Wind speed (m s^{-1})	Wind direction
Oct. 8, 2019 (1st spraying)	1	19.6	76.3	3.0	SW[1)]
	2	19.0	76.8	3.5	SSW[2)]
	3	18.5	77.5	2.4	SSW
	4	18.2	82.8	3.0	SW
Oct. 18, 2019 (2nd spraying)	1	9.8	96.3	-	-
	2	11.2	93.0	-	-
	3	9.5	95.0	-	-
	4	10.8	95.5	-	-
Oct. 28, 2019 (3rd spraying)	1	4.0	94.8	1.2	NNE[3)]
	2	5.5	93.8	-	-
	3	3.9	95.7	-	-
	4	4.7	96.3	-	-

[1)]Southwester, [2)]South-southwester, [3)]North-northeaster.

Table S2. Residues of picarbutrazox in Chinese cabbage 7 days after the last spraying at plot 1.[1)]

Line	Residue (μg kg^{-1})<br>Distance from the starting point of spraying (mean ± SD[2)], n = 3)					
	0 m	20 m	40 m	60 m	80 m	100 m
1	$48^{hi} \pm 2$	$24^{cde} \pm 1$	$18^{bc} \pm 2$	$20^{cd} \pm 1$	$7^{ab} \pm 1$	$17^{abc} \pm 1$
2	$486^{x} \pm 11$	$54^{i} \pm 3$	$105^{kl} \pm 5$	$45^{hi} \pm 4$	$24^{cde} \pm 1$	$176^{r} \pm 6$
3	$127^{no} \pm 8$	$124^{mno} \pm 6$	$141^{p} \pm 5$	$180^{r} \pm 5$	$39^{fgh} \pm 4$	$162^{q} \pm 5$
4	$204^{s} \pm 7$	$41^{fgh} \pm 4$	$19^{bc} \pm 2$	$145^{p} \pm 6$	$143^{p} \pm 1$	$113^{lm} \pm 5$
5	$29^{cdef} \pm 3$	$71^{j} \pm 4$	$42^{gh} \pm 2$	$118^{mn} \pm 1$	$79^{j} \pm 1$	$231^{t} \pm 10$
6	$134^{op} \pm 0$	$292^{u} \pm 19$	$446^{w} \pm 22$	$76^{j} \pm 2$	$402^{v} \pm 17$	$5^{a} \pm 1$
7	$32^{defg} \pm 3$	$25^{cde} \pm 2$	$69^{j} \pm 2$	$112^{lm} \pm 4$	$99^{k} \pm 11$	$35^{efgh} \pm 2$

[1)] The pesticide was diluted 16 times and the multicopter speed was 2 m s^{-1}; [2)] Standard deviation. Different superscript lowercase letters indicate significant differences at $p < 0.05$.

Table S3. Residues of picarbutrazox in Chinese cabbage 7 days after the last spraying at plot 2.[1)]

Line	Residue (μg kg^{-1})<br>Distance from the starting point of spraying (mean ± SD[2)], n = 3)					
	0 m	20 m	40 m	60 m	80 m	100 m
1	5^{a} ± 1	99^{n} ± 5	61^{jk} ± 3	5^{a} [3)] ± 1	29^{cd} ± 2	395^{v} ± 13
2	36^{def} ± 2	53^{hi} ± 3	77^{l} ± 4	114^{op} ± 5	108° ± 2	40^{fg} ± 2
3	137^{r} ± 3	84^{lm} ± 2	45^{gh} ± 1	68^{k} ± 3	33^{def} ± 1	91^{m} ± 7
4	81^{l} ± 5	30^{cd} ± 2	127^{q} ± 2	32^{de} ± 1	31^{de} ± 1	175^{t} ± 5
5	5^{a} [3)] ± 1	23^{bc} ± 1	23^{bc} ± 1	9^{a} ± 2	63^{jk} ± 1	65^{k} ± 4
6	162^{s} ± 17	120^{pq} ± 5	52^{hi} ± 1	7^{a} ± 1	51^{hi} ± 4	38^{efg} ± 3
7	63^{jk} ± 3	82^{l} ± 2	57^{ij} ± 2	124^{q} ± 1	185^{u} ± 2	17^{b} ± 1

[1)] The pesticide was diluted 16 times and the multicopter speed was 3 m s^{-1}; [2)] Standard deviation; [3)] When calculating the average value, an LOQ of 0.005 mg/kg was applied. Different superscript lowercase letters indicate significant differences at $p < 0.05$.

Table S4. Residues of picarbutrazox in Chinese cabbage 7 days after the last spraying at plot 3.[1)]

Line	Residue (μg kg^{-1}) Distance from the starting point of spraying (mean ± SD[2)], n = 3)					
	0 m	20 m	40 m	60 m	80 m	100 m
1	$5^{a} \pm 1$	$42^{f} \pm 2$	$26^{cd} \pm 4$	$11^{a} \pm 1$	$103^{k} \pm 4$	$20^{bc} \pm 1$
2	$81^{hi} \pm 2$	$76^{hi} \pm 1$	$64^{g} \pm 1$	$85^{ij} \pm 5$	$121^{lm} \pm 3$	$182^{q} \pm 8$
3	$285^{t} \pm 13$	$103^{k} \pm 3$	$247^{r} \pm 6$	$148^{o} \pm 9$	$256^{s} \pm 5$	$38^{ef} \pm 1$
4	$91^{j} \pm 6$	$22^{bcd} \pm 0$	$78^{hi} \pm 6$	$80^{hi} \pm 5$	$133^{n} \pm 6$	$9^{a} \pm 1$
5	$316^{u} \pm 9$	$62^{g} \pm 3$	$73^{h} \pm 7$	$115^{l} \pm 5$	$84^{ij} \pm 1$	$31^{de} \pm 2$
6	$126^{mn} \pm 2$	$62^{g} \pm 2$	$124^{m} \pm 3$	$91^{j} \pm 10$	$170^{p} \pm 14$	$14^{ab} \pm 2$
7	$44^{f} \pm 1$	$59^{g} \pm 1$	$8^{a} \pm 1$	$26^{cd} \pm 2$	$36^{ef} \pm 2$	$10^{a} \pm 1$

[1)]Pesticide was diluted 32 times and the multicopter speed was 2 m s^{-1}; [2)] Standard deviation. Different superscript lowercase letters indicate significant differences at $p < 0.05$.

Table S5. Residues of picarbutrazox in Chinese cabbage 7 days after the last spraying at plot 4.[1)]

Line	Residue (μg kg^{-1}) Distance from the starting point of spraying (mean ± SD[2)], n = 3)					
	0 m	20 m	40 m	60 m	80 m	100 m
1	269^{v} ± 3	5^{a} ± 1	20^{cdef} ± 1	48^{j} ± 1	48^{j} ± 4	64^{l} ± 1
2	289^{w} ± 11	23^{efg} ± 1	23^{efg} ± 1	18^{bcde} ± 1	83^{p} ± 4	77° ± 2
3	89^{q} ± 2	68^{lm} ± 2	33^{h} ± 2	70^{mn} ± 1	18^{bcde} ± 1	19^{cdef} ± 1
4	74° ± 5	28^{g} ± 3	17^{bc} ± 1	51^{jk} ± 1	94^{q} ± 2	68^{lm} ± 2
5	160^{u} ± 4	24^{fg} ± 1	22^{def} ± 1	13^{b} ± 1	77° ± 3	74no ± 1
6	17^{bcd} ± 2	40^{i} ± 1	33^{h} ± 1	121^{s} ± 1	143^{t} ± 6	99^{r} ± 4
7	90^{q} ± 3	79^{op} ± 3	18^{bcde} ± 1	55^{k} ± 4	35^{h} ± 3	92^{q} ± 1

[1)]Pesticide was diluted 32 times and the multicopter speed was 3 m s^{-1}; [2)] Standard deviation. Different superscript lowercase letters indicate significant differences at $p < 0.05$.
